# Predicting Selectivity with a Bifurcating Surface: Inaccurate Model or Inaccurate Statistics of Dynamics?

**DOI:** 10.1021/acs.jpca.4c04039

**Published:** 2024-08-05

**Authors:** Kai-Yuan Kuan, Chao-Ping Hsu

**Affiliations:** † Institute of Chemistry, Academia Sinica, 128 Academia Road, Section 2, Nankang, Taipei 11529, Taiwan; ‡ Physics Division, National Center for Theoretical Sciences, 1, Section 4, Roosevelt Road, Taipei 106, Taiwan

## Abstract

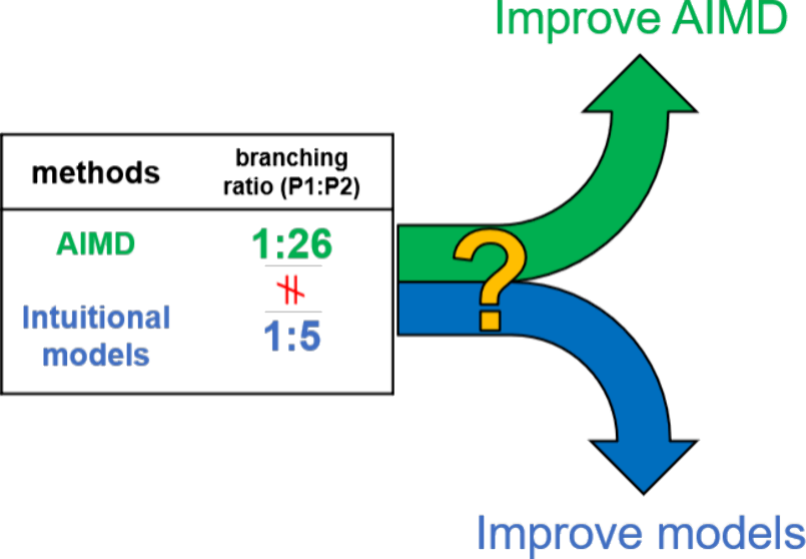

Reactions on post-transition-state bifurcation (PTSB) energy surfaces are an important class of reaction in which classical rate theories, such as the transition state theory, fail to account for the selectivity. Quasiclassical trajectory molecular dynamic (QCT-MD) simulation is an important computational approach to understanding reactions mechanisms, especially for reactions that cannot be predicted from conventional rate theories. However, the applicability of direct dynamic simulations is hampered by huge computational costs for collecting a statistically meaningful set of trajectories, making it difficult to compare simulation results with theoretical or physical insights-based predictions (non-MD predictions). In this work, we examine the PTSB of Schmidt–Aubé reactions studied by Tantillo and co-workers. With machine-learning using kernel-ridge regression (KRR) to predict atomic forces, statistical reliability was enhanced by significantly increasing the number of trajectories. With KRR, the bottleneck of simulating dynamics (atomic forces in QCT-MD with density functional theory) was accelerated more than 100-fold. We found that this KRR-aided QCT-MD approach is successful in predicting branching ratios with a much larger number of trajectories. With our approach, statistical errors are greatly reduced, and hypothetical non-MD models for predicting selectivity are tested with much higher confidence. By comparison with non-MD models, dynamical properties that affect branching ratios become more clearly described.

## Introduction

Correct prediction of reactivity and selectivity is an important task in chemistry. It is also one of the central tasks in computational chemistry, with available approaches ranging from classical rate theories to molecular simulations. Classical rate theories, such as the transition state theory (TST)^1^ or Rice–Ramsperger–Kassel–Marcus (RRKM) theory,^2,3^ are commonly used to predict reaction outcomes. The simple rate dependence on activation energy or free energy furnishes the foundation for insights into many fundamental concepts in reaction rates and selectivity. Despite well-developed theories for reaction rates, there are still numerous cases that cannot be understood and require details at the dynamics level, as seen in many fundamental types of reactions.^4−11^

**Figure 1:**
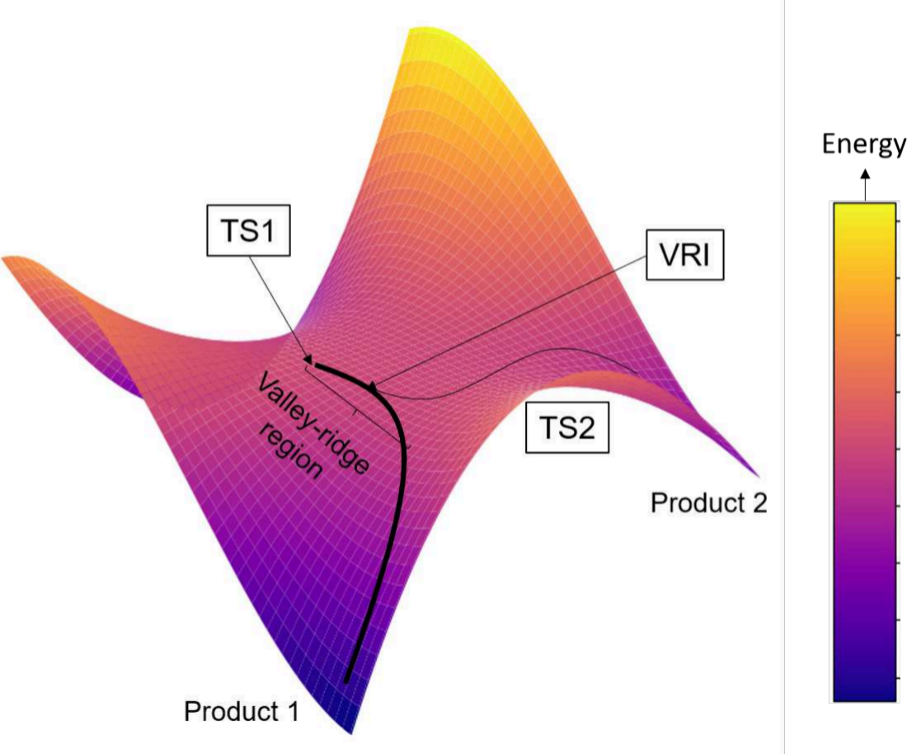
Typical potential energy surface with an asymmetric post-transition-state bifurcation and selectivity favoring product 1. Products are separated by the valley-ridge along which the transition of the two imaginary modes defines two TSs (TS1 and TS2). The valley-ridge inflection (VRI) point occurs where at least one of the Hessian matrix eigenvalues is zero. The surface was generated with the equation *f*(*x*, *y*) = 2*x*^3^ – 5*x*^2^ – 5*xy*^2^ + 3*y*^2^ + *y*^3^ + 2.

An intriguing example is reactions involving a post-transition-state bifurcation (PTSB) energy surface (Figure 1). In this type of reaction, the branching ratio cannot be predicted from energetics of barriers, nor from that of products. Instead, dynamic factors such as atomic velocities in the trajectory *after* passing the first transition state (TS1) are important factors controlling selectivity. Early descriptions of reaction bifurcation were made by Baker and Gill,^12^ followed by Quapp et al., who reported a detailed analysis of bifurcating potential energy surfaces.^13,14^ This idea was also extended to explain selectivity for ordinary organic reactions. In an experimentally and computationally combined work by Houk and Singleton et al.,^15^ the experimental intramolecular kinetic isotope effects (KIEs) of the ene reaction between singlet oxygen and substituted alkenes could be rationalized only by considering a “two-step–no-intermediate” mechanism, in which conventional zero-point energy (zpe) differences between the reactants and the TS failed to account for isotopic selectivity, such that detailed dynamics were required. Since then, reactions with such a PTSB character have been discovered in a wide range of systems, including those important in chemical synthesis^16−18^ and in biological systems.^19,20^

Although PTSB may be important in many systems, identifying the existence of a PTSB surface of a reaction is not an easy task. Mathematically, a bifurcating surface can be characterized by the presence of a VRI point at which one of the eigenvalues of the Hessian matrix is zero.^21,22^ For a symmetrical PTSB surface where the two bifurcated products are identical, the VRI point can be obtained by performing an intrinsic reaction coordinate (IRC)^23,24^ calculation at TS1. For an asymmetric PTSB surface, however, IRC paths from TS1 usually bypass the VRI point and lead to only one of the bifurcated products. Although the presence of a “shoulder” in the IRC path can be qualitatively characterized as the presence of a PTSB surface,^25,26^ other rigorous approaches such as PES construction on different dimensions or dynamic simulations are still needed to validate this hypothesis or to perform quantitative analysis on the branching ratios.

As with any intriguing phenomenon in chemistry, it is always highly desirable to establish predictive hypotheses from chemical or physical principles prior to involvement of molecular dynamic simulation. As an example, in a study by Lee and Goodman,^27^ the selectivity preference can be predicted from the relative direction of the imaginary eigenvector at TS1 (denoted as **a̅** in their work), and branching ratios can be estimated by allowing thermal fluctuation on the other real modes. This was achieved by using a program based on an earlier “Newton” program by Carpenter’s group.^28^ This idea was later extended to reaction systems with shallow intermediates (caldera-type PES) and was termed as the “VRAI” approach.^29^ Later, Srnec and co-workers calculated branching ratios by using the fraction of atomic group kinetic energies of molecules at TS1. This saves effort in obtaining other information, such as TS2 and products, while maintaining good accuracy.^30,31^

Recently, various attempts with machine-learning (ML) have been published. For example, Ess and co-workers tested various ML models in predicting binary classification of PTSB selectivity in some pericyclic reactions,^32,33^ in which characteristics derived from velocities of certain modes have greater importance than other features. In validating such hypotheses or models, running quasiclassical trajectory molecular dynamics (QCT-MD) is the most straightforward and widely acceptable approach for ground-truth dynamic selectivity, and it is critically important to achieve desirable accuracy for selectivity prediction. In this regard, various issues have been discussed extensively in the literature, such as sampling of initial conditions and accuracy of algorithms for propagation trajectories. In the former, a commonly used method is quasiclassical direct molecular dynamics, which start with initial conditions sampled from quantized vibrational mode energy.^10,34^ In the latter issue, accuracy can be determined by the intrinsic accuracy of quantum chemistry calculations for the energies (and forces), the quantum effect of the nuclei, and the adiabaticity of nuclear motions. Typically, for ground-state reactions with large excitation gaps, Born–Oppnheimer approximation without quantum correction is usually accurate enough.^35^

However, it still remains difficult to obtain a suitable statistical margin from QCT-MD trajectories for most known cases of PTSB, since collection of statistically meaningful trajectories can be computationally intensive. As a result, it is always hard to judge whether a discrepancy between the dynamic simulation and a theoretical hypothesis is from an exception to the theory where further improvement is needed or is merely due to insufficient sample size, especially for weakly selective systems. As an illustrative example, it takes over 2500 coin flips to predict the head–tail probability with uncertainty (standard deviation) less than 1%. In determining the branching ratio, which is a very similar binary partition problem, a small error is usually needed when the difference in the probability of forming one product over the other is small. Although such high accuracy is usually not a major pursuit for works involving bifurcated reactions currently, it is still highly desirable when comparing PTSB reactions between subtle changes in experimental parameters, such as solvent or substituent effects.^36^ Therefore, it is necessary to develop efficient approaches for selectivity of PTSB.

Therefore, it is highly desirable to develop an efficient computational scheme while maintaining the advantages of direct dynamic simulation. QCT-MD propagates trajectories with the Verlet algorithm in which the force fields required for generating structures of the next time step are usually the computational bottleneck of the iteration process, if obtained from quantum-mechanical calculations. However, there have been many works applying ML techniques in predicting PES^37^ and force fields,^38^ and dynamical trajectories can be obtained much more efficiently.^39^ Using this approach, physical quantities of interest to chemists, such as vibrational spectra, conformational barriers, and reaction selectivity,^40^ among others, can be efficiently derived. To develop an accurate ML model to predict, it is generally believed that the configurational space spanned by the training examples needs to cover that of ML-predicted samples. In studying reaction dynamics, such training sets can sometimes be large.^41^ However, a study by Zhang et al. demonstrated that the size of the training set can be reduced by limiting the degree of freedom in the reaction space.^41^ This can be achieved by running quasiclassical direct dynamics trajectories initialized at TS and proceeding in the direction of forming products. To the best of our knowledge, an approach using a ML-based force field for reactions with PTSB has not been reported yet. It is thus highly desirable to see how ML assists with statistics of dynamic trajectories in systems with PTSB.

**Scheme 1:**
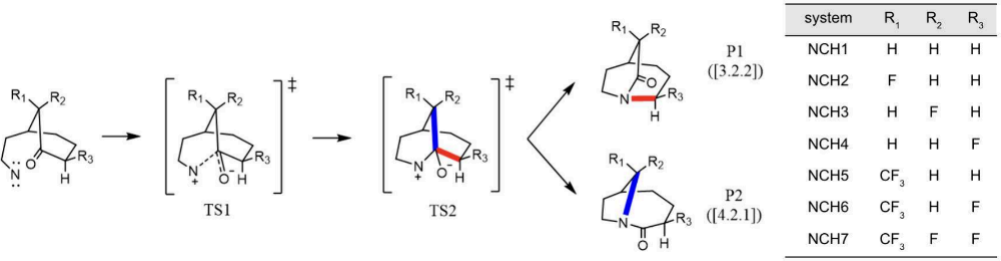
Mechanism of PTSB Schmidt–Aubé Reaction Discussed in ref (26) The two products ([3.2.2] and [4.2.1]) afforded from migration of the red or blue bond are denoted as P1 and P2, respectively.

In this work, we demonstrate that implementation of kernel ridge regression (KRR) in on-the-fly prediction of energy gradients using Coulomb matrices (eq 1) as features in dynamical calculation is a satisfactory approach to analyze reactions with PTSB energy surfaces. Recently, Tantillo and Campos computationally designed Schmidt–Aubé nitrene insertion reactions such that ambimodal transition states are involved.^26^ This was achieved by removing the N_2_ group from the original substrates used in experiments developed by Tani and Stoltz.^42^ Their study provided an intuitive hypothesis, in which dynamics and time, after passing TS1, are related to PTSB selectivity. This correlation was further supported by our previous study using time spent in the VRI region.^43^ We study five systems, NCH*n* (*n* = 1–5), of the modified Schmidt–Aubé nitrene insertion reaction (Scheme 1) from their work. For convenience, the two dynamical products, [3.2.2] and [4.2.1] in Tantillo’s work, are denoted as P1 and P2, respectively. With our implementation, uncertainties about the product branching ratio are greatly reduced by the ability to collect several times more trajectories at significantly reduced computational costs.

## Methods

### General Procedure

**Scheme 2:**
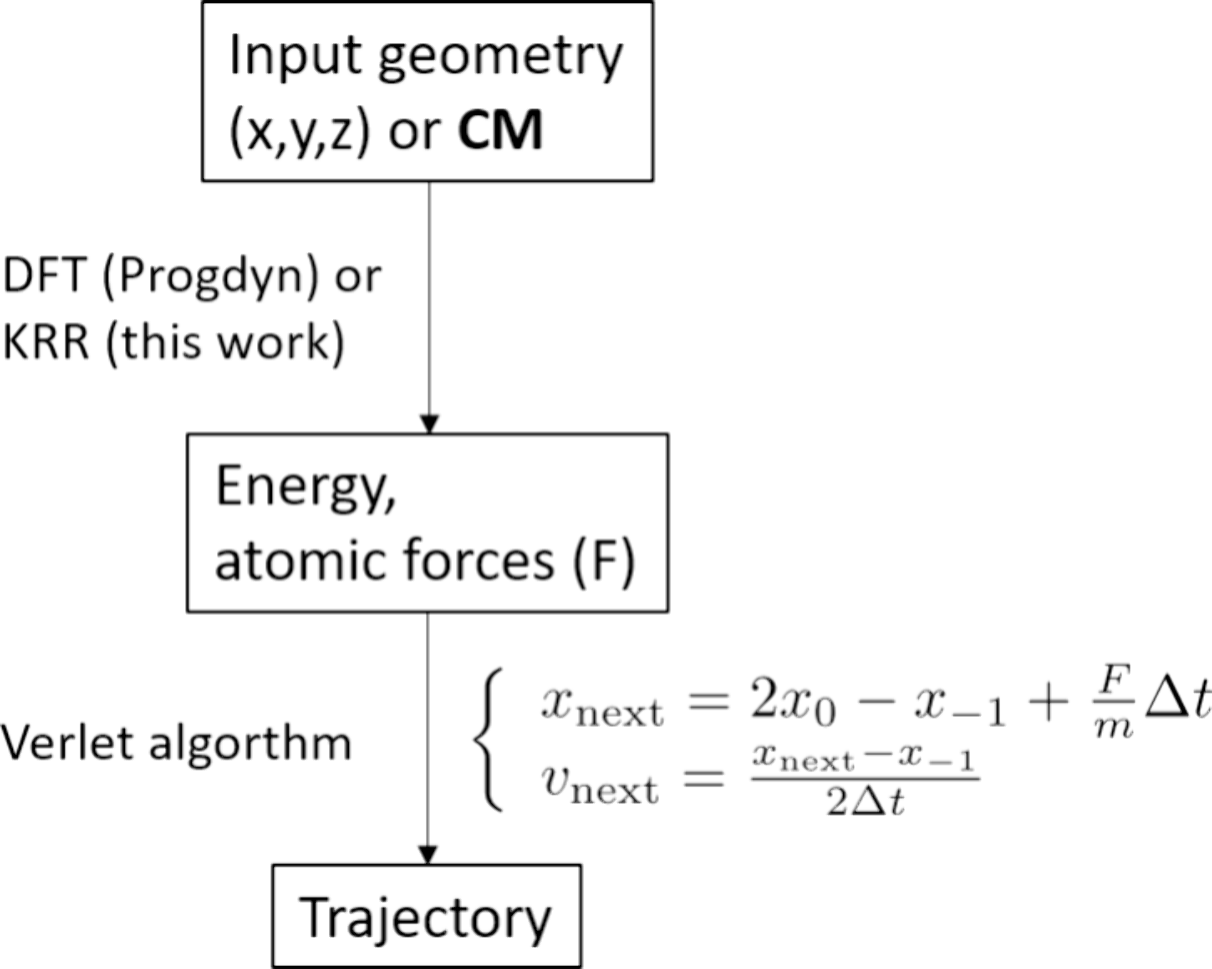
General Procedure for a Progdyn QCT-MD Job Potential energy and atomic forces can be calculated from wave function methods, DFT, or machine-learning (this work).

Calculations of DFT structures, energies, forces, and frequencies employed default procedures in Gaussian16^44^ unless otherwise noted. QCT-MD trajectories were calculated using Progdyn,^45^ and detailed descriptions can be found in the Supporting Information for their recent work^10^ with Gaussian16 used for forces at each point of trajectories. ML-related procedures (including training, testing, and predicting) were performed with Scikit-learn Python packages.^46^ Detailed descriptions and programs used in this work can be found in the Supporting Information. Scheme 2 illustrates the overall workflow of QCT-MD with or without ML, following that of the program suite, Progdyn, developed by Singleton and Oyola,^45^ but the potential energy and atomic forces can be replaced by those derived from ML, when necessary.

### General Procedure for KRR Trajectories

To train KRR models, all structures were converted into Coulomb matrices (CM, **C**), which are a commonly used translationally and rotationally invariant ML feature (eq 1).^47^ Since the CM is a symmetric matrix, only the lower triangular elements were taken and reordered as a one-dimensional array as input features. ML algorithms were then optimized by minimizing the loss compared to true (DFT-calculated) quantities. These trained KRR models were implemented in Progdyn, such that the subroutine program (*progdynb*) for integrating trajectories was switched into KRR procedures. That is, the initial structure and velocity for the QCT-MD and KRR trajectory were both sampled quasi-classically and generated the first two points from DFT methods. Starting from the third point, the atomic force in the Verlet algorithm^48^ was predicted from trained KRR models at given structures on-the-fly. KRR algorithms were imported from the Scikit-learn package without implementing other plugins or packages from other sources.^46^
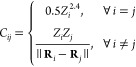
1

### Training the KRR Model

To train the KRR model, all data sets were randomly divided into five sets for fivefold cross validation, in which four sets served as training data and the remaining one was reserved for testing. These data points were shuffled and fed into KRR algorithms obtained from the Scikit-learn Python package.^46^ KRR algorithms used in this work can be expressed in eqs 2–4. Using radial basis function (rbf) kernels, hyperparameters (variance (σ^2^) and regularization (λ)) were optimized using GridSearchCV in the Scikit learn library. (Detailed settings, such as the ratio of training and testing sets and hyperparameters, are included and discussed in the Results and Discussion section.) After obtaining the lowest loss calculated by eq 4, weighting factors (α) corresponding to the best (σ, λ) were then used to predict new trajectories.^49^
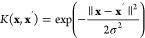
2
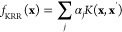
3
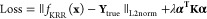
4

### Parameters for QCT-MD Trajectory Calculation

In all QCT-MD calculations included in the present work, initial structures around TS1 (see Scheme 1) were sampled quasi-classically by setting “initialdis 2” in the subprogram *progdyn.conf* of the Progdyn package. The direction of initial velocity followed the imaginary mode toward product. Trajectories initiated from both forward and reverse directions of the imaginary mode were sampled. The trajectory was then propagated based on DFT-calculated interatomic forces using the Verlet algorithm. A thermostat was implemented by introducing a small damping coefficient (0.99–1) to the velocity term, which was chosen to reach the lowest error in total atomic kinetic energy compared with QCT-MD trajectories (further details in the Supporting Information). Trajectories were integrated with 1 fs increments and terminated when either of the two dashed bonds in Scheme 1 was below 1.7 Å or reached the maximum number of points, which was set to 350 fs.

## Results and Discussion

### Performances of the KRR Models

**Figure 2:**
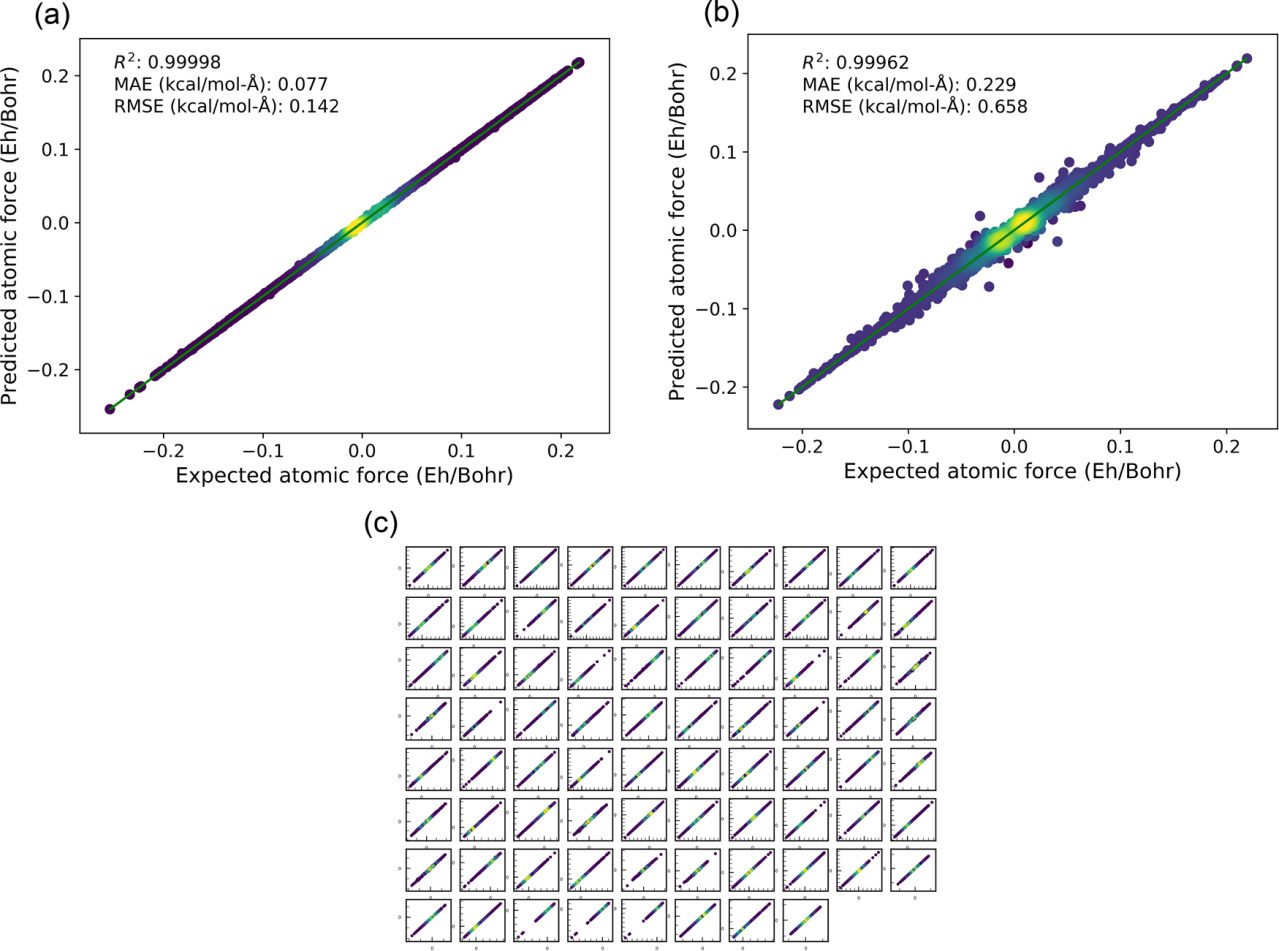
Parity plots for (a) training, (b) testing for all the vector components in the forces, and (c) testing on each individual atomic force vector component of the NCH5 system. Starting from top left corner, moving horizontally first, the subplot corresponds to the atomic force in the *x*-direction of the first atom, *y*-direction, and *z*-direction, followed by those of the second atom, and so on. Each minor tick mark represents a 0.02 E_h_/Bohr increment with respect to the origin labeled with major tick marks. The *R*^2^, MAE, and RMSE represent the squared correlation coefficient, mean average error, and root-mean-square error, respectively. The yellow regions represent high-density data points, and blue, low.

As the first attempt, we picked the NCH5 system to train the KRR model since it was reported to have smallest selectivity (49.1%) in Tantillo’s work.^26^ A set of QCT-MDs containing 452 trajectories containing 60 746 points of which 149 (33.0%) formed P1 were collected. Figure 2 shows the parity plots on the training and testing set for the NCH5 system with the procedure described in the Methods. It turns out that CM is an appropriate ML feature in predicting atomic forces as the overall errors (MAE and RMSE) are well below the chemical accuracy (∼1 kcal/mol-Å).

To further test the performance of KRR models in predicting trajectories with correct outcomes, Table 1 shows the confusion matrix for predicting the NCH5 system given its initial geometry and velocities, taken from 191 QCT-MD trajectories that lead to one of the products. The two diagonal elements represent trajectories in which the KRR models correctly predicted what QCT-MD got, whereas the off-diagonals are for those either predicted incorrectly or that became divergent. The overall correct rate exceeds 95%, indicating that this KRR-aided QCT-MD is successful in predicting the branching ratio. This is also comparable to or even better than other common classification-type ML algorithms, which range from 56% to 83%.^33^

**Table 1:** Confusion Matrix of the NCH5 System for Predicting Outcomes of 191 QCT-MD Trajectories Given Their Initial Conditions (Geometry and Velocity)

		QCT-MD	
		P1	P2
	P1	61	6
KRR	P2	2	121
	X^a^	1	0

a Undefined results of trajectories that either stayed as intermediates at the time limit or diffused beyond space defined by the training data.

Having tested the accuracy of the KRR model, new trajectories can be collected using KRR-predicted force embedded in the QCT-MD routine. Such KRR-aided QCT-MD was performed, and a much larger set containing 2824 trajectories was collected in which 919 (32.6%) of them formed P1. This is very close to the branching ratio of the QCT-MD data used to train this model (33.0%). However, the closeness of branching ratios between the original QCT-MD used for training and the corresponding KRR-aided QCT-MD causes concern if the branching ratio of the latter depends on the composition of the former. To test this, three sets of trajectories with different branching ratios—(1) 1:2, (2) 1:1, and (3) 2:1—were used to train the KRR models, and the results are summarized in Table 2.

**Table 2:** Selectivity of KRR Trajectories Trained by AIMD with Different Compositions of Products for the NCH5 System

trajectory composition of training data (P1:P2)	number of total QCT-MD trajectories^a^	selectivity of KRR trajectories, P1:P2 (P1/(P1 + P2))
1:2	452	919:1905 (0.325)
1:1	200	212:736 (0.22)
2:1	190	162:490 (0.25)
1:1	56^b^	111:188 (0.37)^c^

a Total number of QCT-MD trajectories collected to optimize the KRR model, including training, testing, and validation set.

b Same trajectory data as that reported in Tantillo’s work.

c Determined by if one of the colored bonds shown in the TS2 in Scheme 1 is greater than 1.7 Å (red: P1, blue: P2).

In all cases, we found the branching ratios slightly favor P2. In addition, this is consistent even when more trajectories affording P1 were included in the training data set. The insensitivity of branching ratios with respect to composition of training data shows the robustness of KRR-aided QCT-MD in predicting branching ratios. Finally, using the same set of trajectories as in Tantillo’s work did not give productive trajectories due to the insufficiency of training data. Accordingly, predicted trajectories were mostly divergent. However, the ratio of trajectories reaching the dynamic intermediate of P1 and P2 (judged by the bond cleavage of the colored bond at the TS1 in Scheme 1) still shows that P1 is less favored (0.37). This supports the accuracy of the branching ratios predicted from the KRR-aided QCT-MD approach.

### Branching Ratios from KRR-Aided QCT-MD on Other Systems

Having validated the branching ratios of the NCH5 system predicted from the KRR-aided QCT-MD, we further explored whether this method still provides good predictions on other similar systems. Table 3 summarizes the results of KRR-aided QCT-MD branching ratios for the NCH1–5 systems. It shows that the KRR-aided QCT-MD trajectories mostly reproduce the branching ratios of QCT-MD trajectories of four more systems reported in Tantillo’s work. Besides the NCH5 system, a relatively large deviation is seen in the NCH3 system. However, by collecting a much larger set of QCT-MD trajectories, we found that only 13 (6.3%) of trajectories formed P1 from a total of 206 trajectories. This is again closer to that predicted from KRR-aided QCT-MD trajectories.

**Table 3:** Comparison of Dynamic Trajectories and Branching Ratios Predicted Using Non-MD Models

		QCT-MD^a^		KRR-aided QCT-MD			non-MD models	
system	ΔΔ*G*^b^	no. traj.^c^	P1 (%)	no. traj.^c^	P1 (%)	avg. traj. time (fs)	VRAI^d^	RMCF^e^
NCH1	5.2	75	4.0	1136	7.7	72.9	3.6	18.7
NCH2	–2.8	115	27.0	847	24.4	78.4	29.9	21.0
NCH3	–6.0	79	16.5	986	2.0	83.5	3.7	15.7
NCH4	–3.5	76	1.3	2091	3.5	76.8	0	10.5
NCH5	–6.5	57	49.1	2824	32.6	84.9		

a From ref (26).

b P2–P1 in kcal/mol.

c Only trajectories ended up as one of the products are counted.

d From ref (27).

e From ref (31).

### Comparison with Non-MD Models

Recently, Goodman,^27^ Srnec,^31^ and co-workers developed two models to predict branching ratios from non-MD approaches. To calculate branching ratios, the former model considers geometrical parameters on PTSB surfaces from a four-point (TS1, TS2, P1, and P2) input. (This is later generalized as the Valley Ridge Augmented Implementation (VRAI) model^29^), whereas the latter model considers the kinetic energy fraction at the TS1^30^ (called reaction mode composition factor (RMCF) analysis).

Prediction results using these two models on NCH1–4 systems are also listed in Table 3. Comparing with the KRR-aided QCT-MD results in this work, we note that the seemingly larger deviation in the VRAI model on the NCH3 system is greatly reduced if compared with QCT-MD containing more trajectories. On the other hand, compared with those by RMCF, the trend of model prediction still largely holds. However, the discrepancy was increased by comparing KRR-aided QCT-MD results. Despite the high accuracy in the original report in the NCH3 system, such accuracy is not consistent for other systems with much higher selectivity (NCH1 and NCH4). This shows an example that the inclusion of later points on the PES (TS2 and PDs) may be helpful in increasing the accuracy of non-MD models.

**Figure 3:**
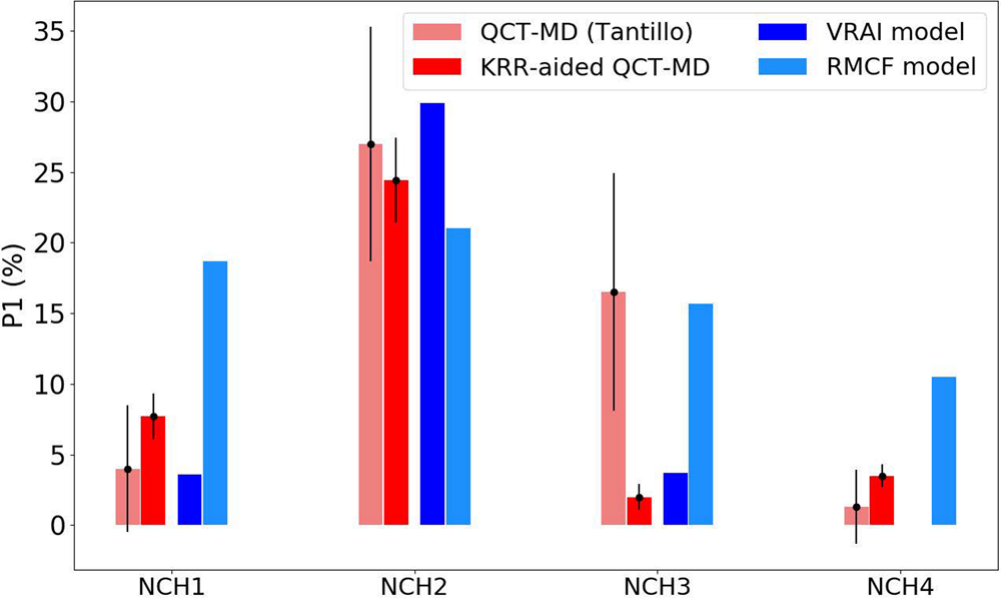
Branching ratios based on P1 (%) predicted by full AIMD (red), KRR-aided QCT-MD (green, this work), VRAI model (purple), and RMCF model (gray). The error bars indicate the 95% confidence interval calculated from binomial statistics (, where *p* is the branching ratio).

Figure 3 summarizes the branching ratios predicted by AIMD approaches and the two intuitive models (VRAI and RMCF) introduced here. While the other systems fall within the confidence intervals of the full AIMD, the NCH3 shows a significant deviation from the full AIMD result despite having relatively shorter |**g̅**| (<0.5) and larger ϕ (>50°). Systems with these parameters tend to have lower errors. However, this problem can be solved by collecting more trajectories using KRR-aided QCT-MD. The closer agreement between the KRR-aided QCT-MD and RMCF model implies that this system may require more stationary points on the PES in order to give accurate predictions on the branching ratios.

Since a PTSB surface is usually exothermic, branching ratios depend on trajectory paths rather than thermodynamical properties. This is usually referred to dynamic matching effect or ”memory effect”.^10,50,51^ This occurs when the lifetime of the dynamical intermediate is too short to complete intramolecular vibrational relaxation (IVR), which would lead to the product valley in the direction of momenta.^5,52^ For such reactions, the lifetime of dynamical intermediates would show a significant effect in controlling selectivity compared to energetics of the PES. For example, in Tantillo’s work, there exists a correlation between the time spent by the trajectories leading to P2 and P1 ratios.^26^ The longer the lifetime of trajectories, the higher chance they would fall into the other product valley. In contrast, the relative free energies between the two products cannot account for the selectivity observed in QCT-MD. The NCH1 should have mostly P1 whereas the other four systems should have mostly P2, which is not the case.

**Figure 4:**
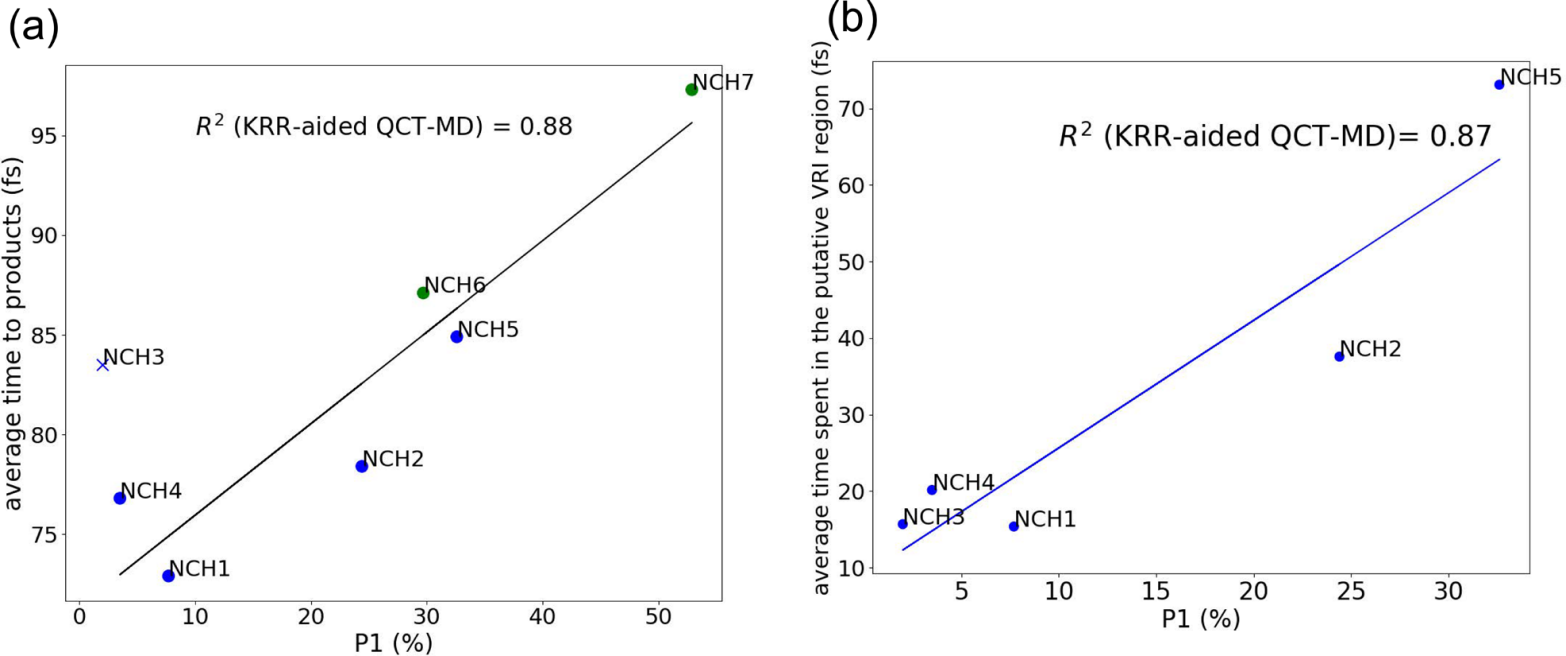
(a) Correlation of the product forming time and the P1 ratio of the extended sampling of trajectories in this work. *R*^2^ = 0.88 (not considering NCH3, blue cross). For NCH6 and NCH7, only QCT-MD data were obtained. Blue dots denote data obtained from KRR-aided QCT-MD, and green dots represent data obtained from extensive sampling of QCT-MD (see Table 3 and the Supporting Information). (b) Correlation of time spent in the putative VRI region calculated from ref (43) and the P1 ratio of the KRR-aided QCT-MD in this work. *R*^2^ = 0.87 (including NCH3).

Using results obtained from the KRR-aided QCT-MD, such correlation is still roughly present: the NCH5 system (84.9 fs) affords more P1 and has much longer average trajectory time than that in NCH1,2,4 systems (72.9–78.4 fs), which afford exclusively P2. Table 3 This is also supported by the extensive collection of QCT-MD trajectories (see the Supporting Information). In addition, the linear correlation between the branching ratio and the average time of productive trajectories becomes much stronger in this work with many more trajectories included (*R*^2^ = 0.86 as compared to 0.68 reported in Tantillo’s work, Figure 4).^53^ This shows that obtaining a large number of trajectories, as with any other statistical problem, is helpful in drawing conclusions about mechanistic hypotheses, and the resulting computational costs can be mitigated by implementation of the KRR algorithms.

There exists an outlier system (NCH3), which does not follow the trend predicted by this time correlation. This shows that a simple analysis of total length trajectories may not truly reflect the lifetime of the dynamical intermediate that controls the branching ratios. In our previous work, we developed a more systematic approach that showed that the time spent in the putative VRI region is comparatively shorter than NCH1 and NCH4, which are systems with exceptionally high selectivity favoring P2.^43^Figure 4b shows that the abnormality of NCH3 is eliminated if time spent in the putative VRI time is considered. That is, the apparent longer trajectory time of the NCH3 system is because it actually takes less time in regions that are important in determining the selectivity. This provides a potential perspective in controlling PTSB selectivity, which will be one of our future studies.

## Conclusion

In this work, we addressed the issue of an insufficient number of trajectories in benchmarking prediction of nondynamical models. By introducing KRR algorithms, we reduced computational costs while maintaining the desired accuracy for quantities of interest to chemists. Our results show that KRR-aided QCT-MD is robust and can be used to provide accurate predictions on dynamic selectivity. In addition, we found that branching ratios calculated from KRR-aided QCT-MD are not sensitive to composition of trajectories in the training set. These results enable efficient analysis of dynamical behaviors in reactions with PTSB surfaces. We further demonstrated that Goodman’s VRAI approach is a useful approximation for this system, and the seemingly larger deviations in some cases are reduced by considering larger sets of dynamical results. Finally, our results demonstrate with higher confidence that the time spent by the productive trajectory, or the time in the putative VRI region, can be another useful approach in predicting, or even controlling, branching ratios of PTSB reactions.

## Supporting Information Available

Computational procedures with detailed descriptions and programs used in this work; QCT-MD trajectories; parity plots for training and testing on the atomic force of NCH1–NCH3 (PDF)
