# Supplementary material for: Predicting Selectivity with a Bifurcating Surface: Inaccurate Model or Inaccurate Statistics of Dynamics?
Source: J Phys Chem A. 2024 Aug 5;128(32):6798–805. doi: 10.1021/acs.jpca.4c04039 (PMC11331512; doi:10.1021/acs.jpca.4c04039)
Supplement: Supplementary file 1 — jp4c04039_si_001.pdf [file jp4c04039_si_001.pdf]

Supporting Information: Predicting Selectivity with Bifurcating Surface: Inaccurate Model or Inaccurate Statistics of Dynamics?

Kai-Yuan Kuan† and Chao-Ping Hsu*,†,‡

†*Institute of Chemistry, Academia Sinica, 128 Academia Road, Section 2, Nankang, Taipei 11529, Taiwan*

‡*Physics Division, National Center for Theoretical Sciences, 1, Section 4, Roosevelt Road, Taipei 106, Taiwan*

E-mail: cherri@sinica.edu.tw

Computational Procedures

KRR-aided QCT-MD

The optimal kernel-ridge regression (KRR) models used in this work was obtained in the following procedure:

(1) A standard quasiclassical trajectory molecular dynamics (QCT-MD) starting from the optimal TS1 was run to collect a set of trajectories. This was achieved using the Progdyn package developed by Singleton and coworkers.

(2) The molecule geometries, originally in Cartesian coordinate, are converted into Coulomb matrices ($\mathbf{C}$). Their lower triangular part was taken and reshaped into a one-dimensional feature.

(3) The KRR hyperparameters, σ and λ, shown in the Eq. (2) and Eq. (4) in the main text are optimized using the `GridSearchCV` package from Scikit-learn Python packages.

(4) The hyperparameters obtained from (3) are used to generate the weighting factor, α, shown in the Eq. (3) of the main text.

(5) The optimal KRR model obtained from step (3) to (4) are used to predict the atomic forces in place of the quantum-mechanical calculations in the subroutine of Progdyn.

The trajectories of the obtained from original KRR model suffer from early termination due to error propagation. One solution to this problem is by implementing the so-called damping coefficient during the propagation of trajectories. This can be achieved by adjusting damping parameter in *progdyn.conf* of the *Progdyn* for AIMD trajectories. The same option was also implemented in the KRR trajectories. Figure S1 shows an example for the time progression of the total atomic kinetic energy under different damping coefficients for one of the AIMD trajectories. It can be found that a good thermostat behavior could be obtained by implementing a small damping coefficient (df = 0.993 in this case). However, it was found that selectivity of the classification was not affected significantly compared to the undamped trajectories (df = 1).

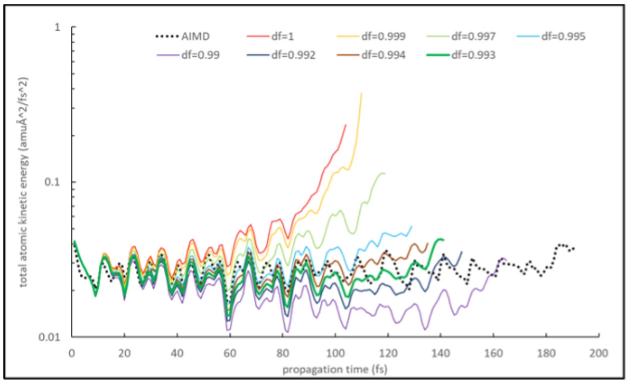

Figure S1: Progression of total atomic kinetic energy of KRR trajectories under different damping coefficients from 0.990 to 1.000 with 0.001 increment (colored solid curves), compared with the original AIMD trajectory (black dotted).

Composition of training set and the hyperparameters of the KRR models

The hyperparameters used in the KRR models and the corresponding performances are summarized in Table S1. Figure S2 to Figure S6 shows the parity plots for each systems for training and testing sets, and Figure S7 to Figure S11 shows the parity plot for each vector element of each system.

Table S1: Information of KRR models used to predict branching ratios. The training set and testing set contains 80% and 20% of the total data respectively. The training and testing errors are in kcal/mol-Å.

		Hyperparamters		Training errors		Testing errors	
System	Total number of data points	σ	λ	MAE	RMSE	MAE	RMSE
NCH1	$51,520$	29.8	1.64×10^{-9}	0.075	0.17	0.30	0.81
NCH2	$51,919$	22.4	3.16×10^{-10}	0.0068	0.014	0.12	0.59
NCH3	$65,241$	14.1	2.15×10^{-8}	0.022	0.028	0.15	0.27
NCH4	$80,000$	70.7	1.00×10^{-10}	0.15	0.032	0.35	0.80
NCH5	$66,000$	44.7	2.50×10^{-9}	0.064	0.118	0.22	0.47

(a)

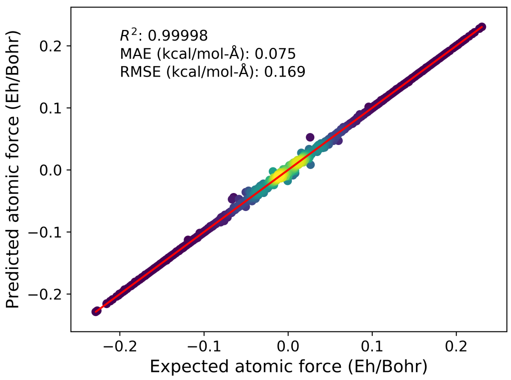

(b)

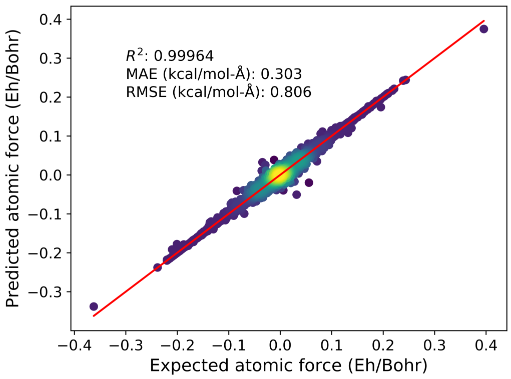

Figure S2: Parity plots for (a) training, and (b) testing on the atomic force of NCH1. The R^2, MAE, and RMSE represents the squared correlation coefficient, mean average error, and root-mean-square error.

(a)

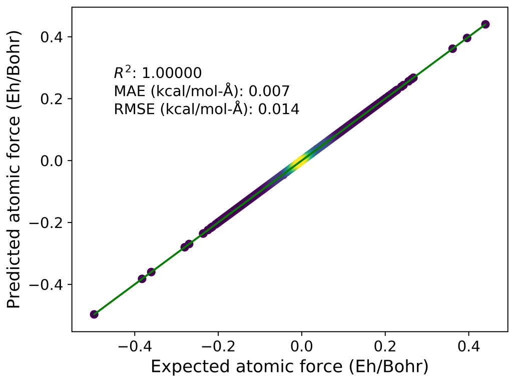

(b)

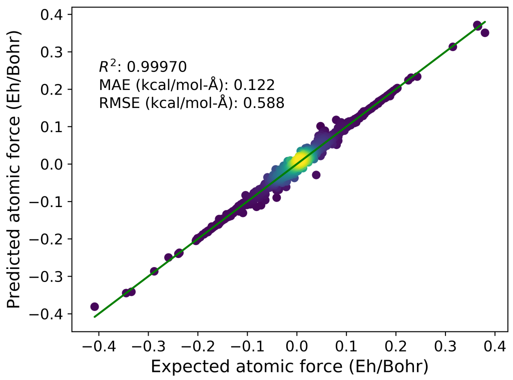

Figure S3: Parity plots for (a) training, and (b) testing on the atomic force of NCH2. The R^2, MAE, and RMSE represents the squared correlation coefficient, mean average error, and root-mean-square error.

(a)

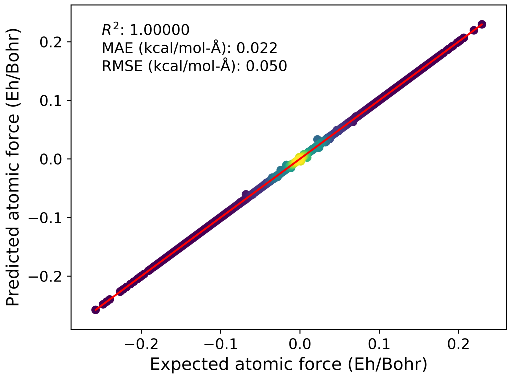

(b)

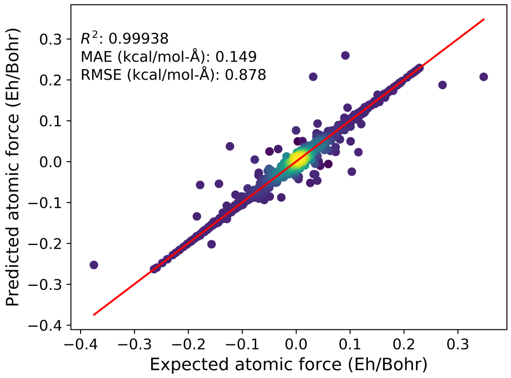

Figure S4: Parity plots for (a) training, and (b) testing on the atomic force of NCH3. The R^2, MAE, and RMSE represents the squared correlation coefficient, mean average error, and root-mean-square error.

(a)

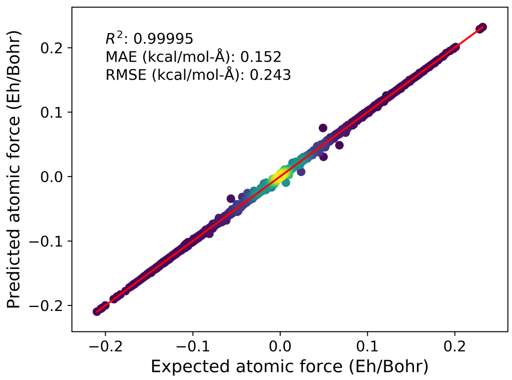

(b)

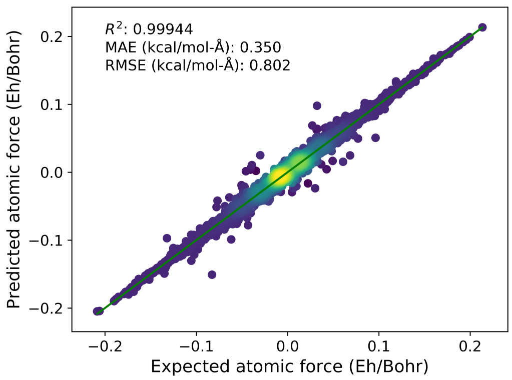

Figure S5: Parity plots for (a) training, and (b) testing on the atomic force of NCH4. The R^2, MAE, and RMSE represents the squared correlation coefficient, mean average error, and root-mean-square error.

(a)

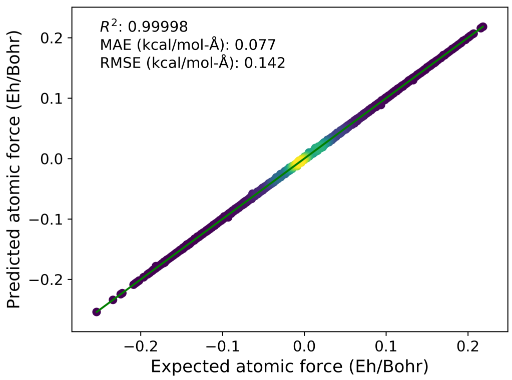

(b)

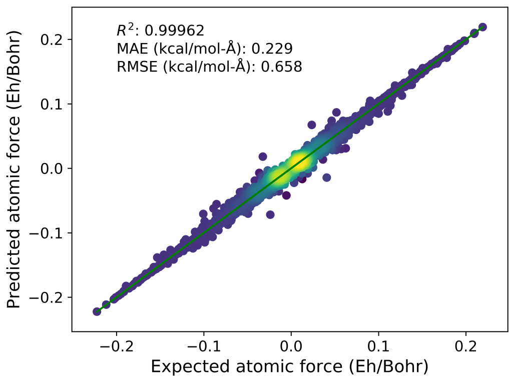

Figure S6: Parity plots for (a) training, and (b) testing on the atomic force of NCH5. The R^2, MAE, and RMSE represents the squared correlation coefficient, mean average error, and root-mean-square error.

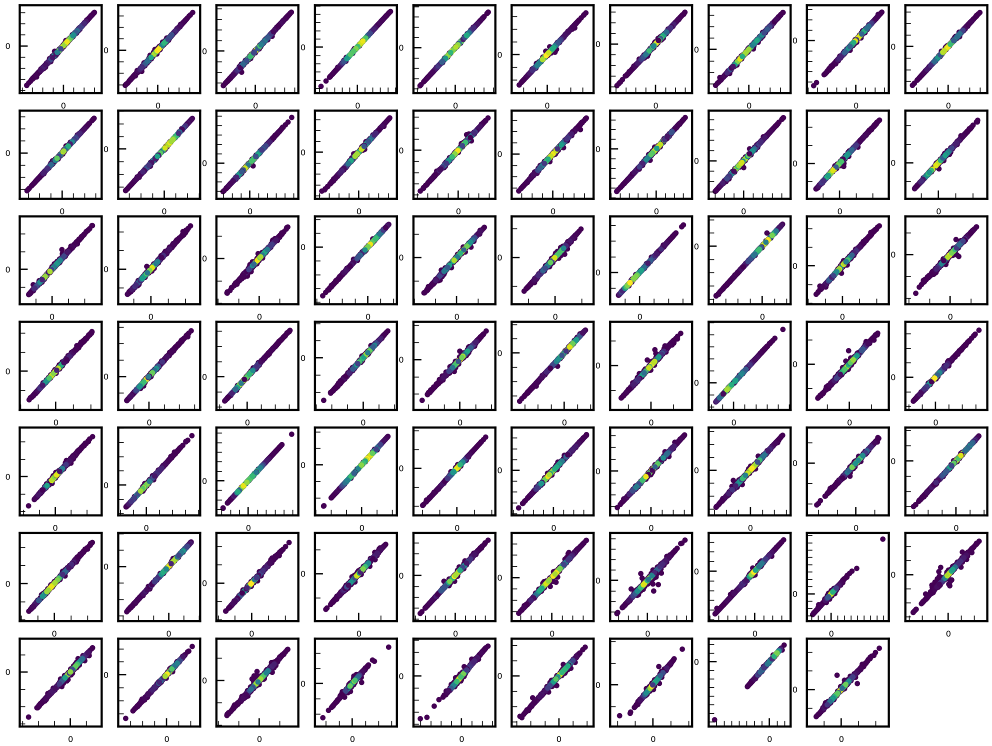

Figure S7: Test set performance of KRR predicted forces on each force vector component on each coordinate of NCH1 system. Starting from the upper left corner and moving to the right, the first plot presents a correlation scatter plot of predicted force vs. DFT force of the first atom, first coordinate (X), and then the first atom, second coordinate (Y), and so on. Higher data-point-density areas are in brighter colors from deep blue to bright yellow. Each subplot tick mark, relative to labelled 0, represents a 0.05 E_h/bohr increment.

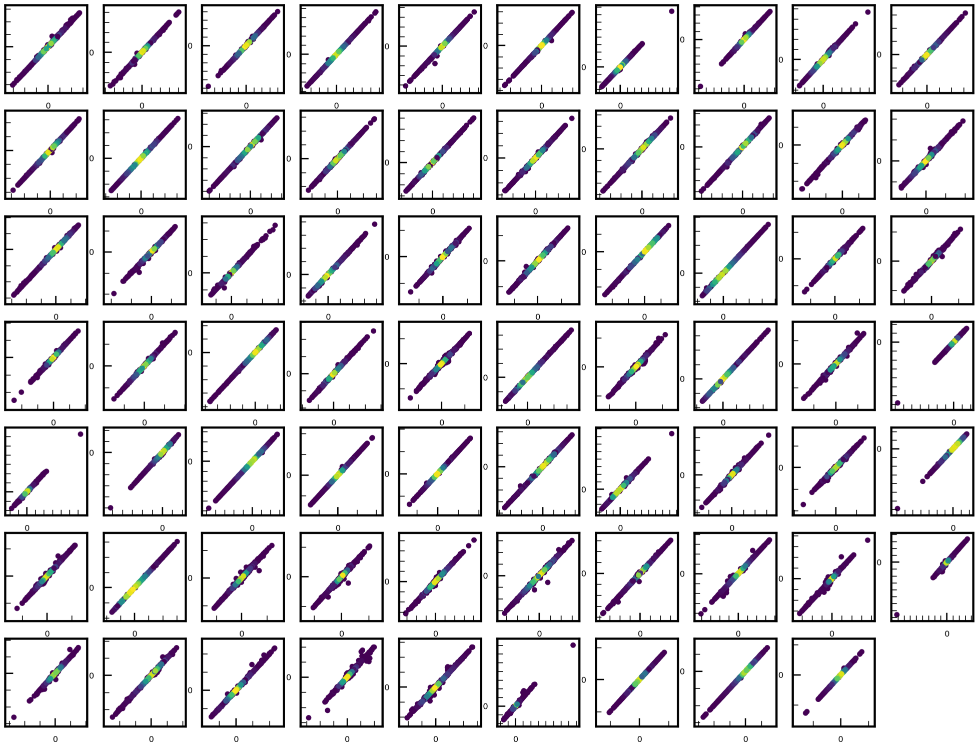

Figure S8: Test set performance of KRR predicted forces on each force vector component on each coordinate of NCH2 system. Starting from the upper left corner and moving to the right, the first plot presents a correlation scatter plot of predicted force vs. DFT force of the first atom, first coordinate (X), and then the first atom, second coordinate (Y), and so on. Higher data-point-density areas are in brighter colors from deep blue to bright yellow. Each subplot tick mark, relative to labelled 0, represents a 0.05 E_h/bohr increment.

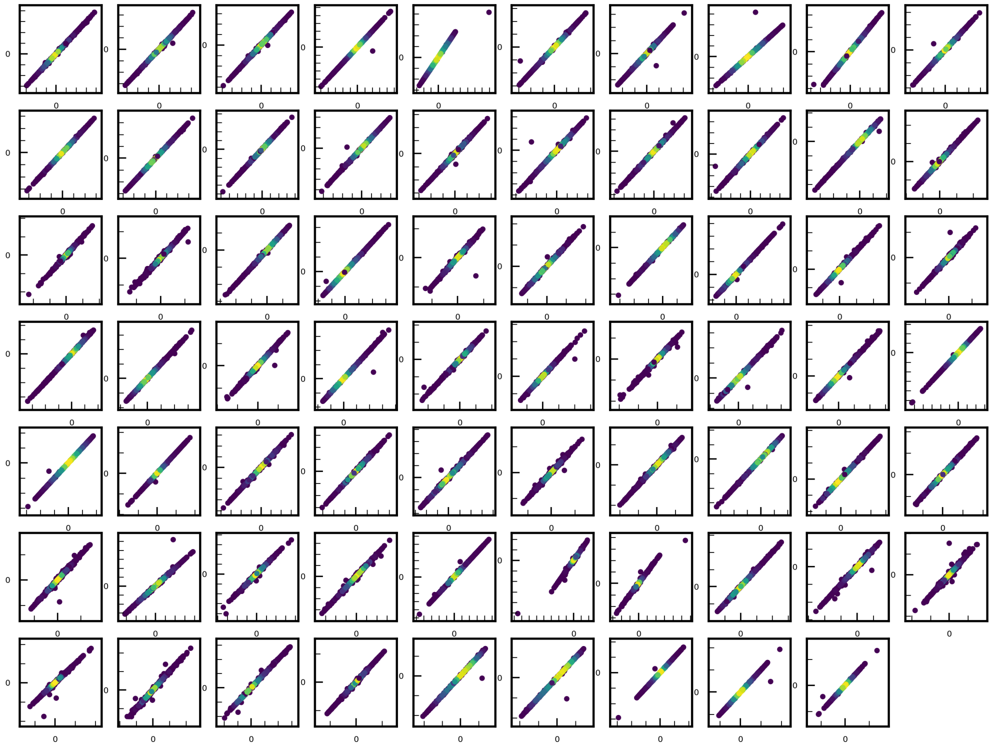

Figure S9: Test set performance of KRR predicted forces on each force vector component on each coordinate of NCH3 system. Starting from the upper left corner and moving to the right, the first plot presents a correlation scatter plot of predicted force vs. DFT force of the first atom, first coordinate (X), and then the first atom, second coordinate (Y), and so on. Higher data-point-density areas are in brighter colors from deep blue to bright yellow. Each subplot tick mark, relative to labelled 0, represents a 0.05 E_h/bohr increment.

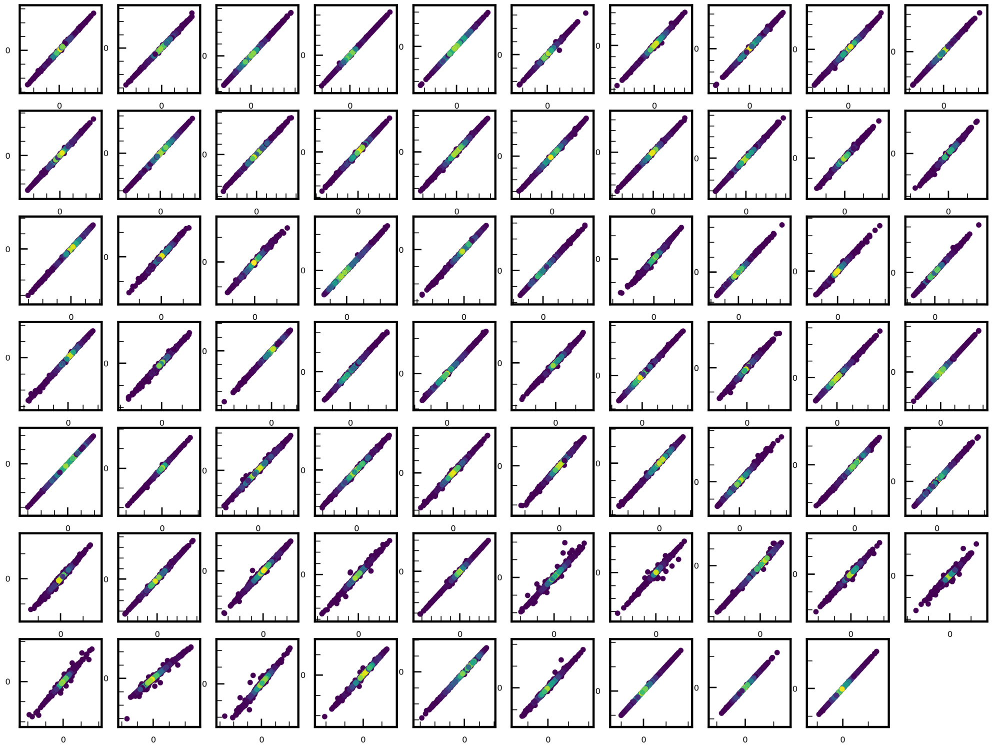

Figure S10: Test set performance of KRR predicted forces on each force vector component on each coordinate of NCH4 system. Starting from the upper left corner and moving to the right, the first plot presents a correlation scatter plot of predicted force vs. DFT force of the first atom, first coordinate (X), and then the first atom, second coordinate (Y), and so on. Higher data-point-density areas are in brighter colors from deep blue to bright yellow. Each subplot tick mark, relative to labelled 0, represents a 0.05 E_h/bohr increment.

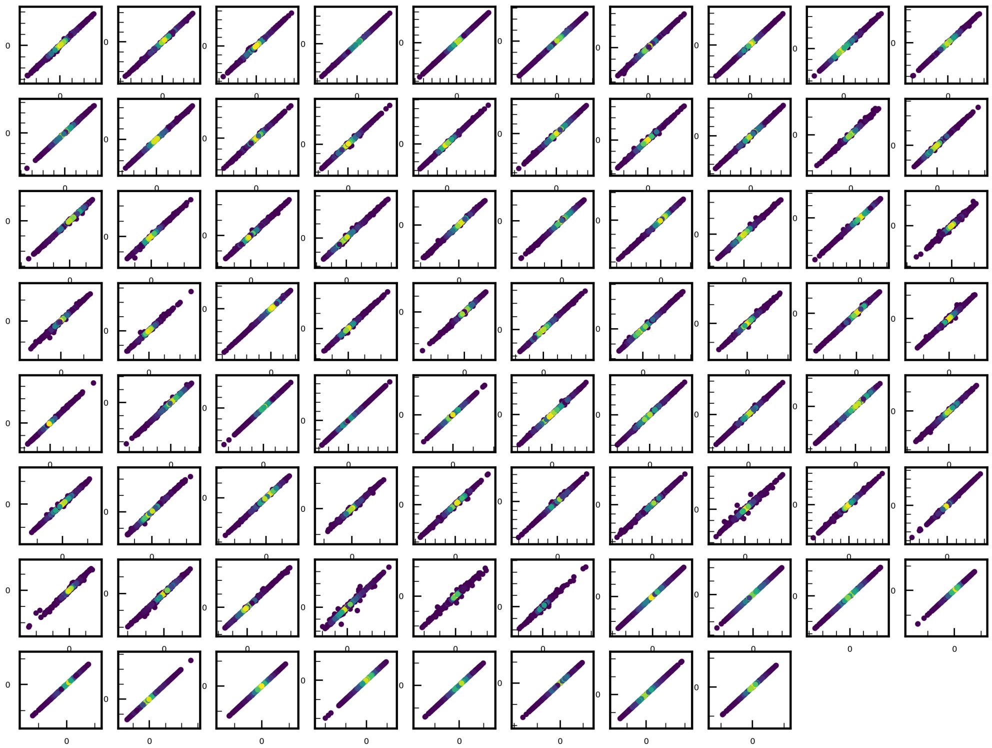

Figure S11: Test set performance of KRR predicted forces on each force vector component on each coordinate of NCH5 system. Starting from the upper left corner and moving to the right, the first plot presents a correlation scatter plot of predicted force vs. DFT force of the first atom, first coordinate (X), and then the first atom, second coordinate (Y), and so on. Higher data-point-density areas are in brighter colors from deep blue to bright yellow. Each subplot tick mark, relative to labelled 0, represents a 0.05 E_h/bohr increment.

Branching ratios from extensive collection of QCT-MD

To validate the branching ratios predicted from the KRR models, Table S2 shows results of additional QCT-MD trajectories of NCHn (n = 1-7) collected in this work. Our results on the P1 ratios mostly agree with those reported in Tantillo's work, but with many more trajectories for each system. Larger deviations can be found in the NCH3 and NCH5 systems. In addition, the time spent in the VRI region, which is approximated as the average time of

all the productive trajectories is also found to be correlated with the selectivity except NCH3. Despite such deviations, it was found that the time correlation with branching ratios become stronger as shown in Figure S12 This indicates that an extensive collection of trajectories helps to make some mechanistic properties more clearer. The outlier problem of the NCH3 case can be resolved by using the time spent in the putative VRI region, which is discussed in the main text as well as in our previous work.[1]

Table S2: Comparison of QCT-MD results used to validate the KRR-aided QCT-MD results with extensive collection of trajectories with those reported by Tantillo and coworkers.

	This work				Data from reference[2]		
System	Number of trajectories[a]	P1 ratio (%)[b]	average time of P2 trajectories (fs)	average time of *all* productive trajectories (fs)	Number of trajectories[a]	P1 ratio (%)[b]	average time of P2 trajectories (fs)[d]
NCH1	631	2.5	62.1	62.9	75	4.0	65
NCH2	371	19.6	72.4	76.0	115	27.0	73
NCH3	470	6.3	84.8	85.3	79	16.5	86
NCH4	706	3.5	73.1	73.1	76	1.3	69.5
NCH5	444	33.5	76.3	79.4	57	49.1	75.7
NCH6	527	29.7	78.8	87.1	47	29.8	75.2
NCH7	473	52.9	90.8	97.3	46	56.5	90.7

[a] Including unproductive trajectories
[b] P1 ratio = P1 trajectories / (total productive trajectories) = P1 / (P1 + P2)
[c] Total number of trajectories successfully crossed TS1.
[d] Approximated from points in Figure 7. of their work.

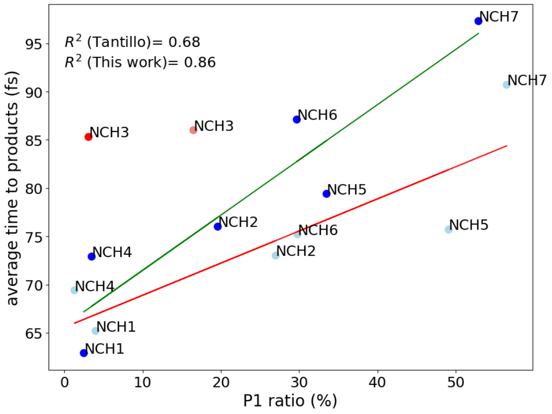

Figure S12: Comparison of QCT-MD results of ref 2 (light-blue dots and red line) and this work (blue dots and green line). The extensive collection of trajectories improves time correlation with P1 ratios. The NCH3 are not included in the regression line (red: this work, light-red: ref. 2).

References

(1) Chuang, H.-H.; Tantillo, D. J.; Hsu, C.-P. Construction of Two-Dimensional Potential Energy Surfaces of Reactions with Post-Transition-State Bifurcations. *J. Chem. Theory Comput.* **2020**, *16*, 4050–4060.

(2) Campos, R. B.; Tantillo, D. J. Designing Reactions with Post-Transition-State Bifurcations: Asynchronous Nitrene Insertions into C–C σ Bonds. *Chem* **2019**, *5*, 227–236.
